# Enhancing generalization in a Kawasaki Disease prediction model using data augmentation: Cross-validation of patients from two major hospitals in Taiwan

**DOI:** 10.1371/journal.pone.0314995

**Published:** 2024-12-31

**Authors:** Chuan-Sheng Hung, Chun-Hung Richard Lin, Jain-Shing Liu, Shi-Huang Chen, Tsung-Chi Hung, Chih-Min Tsai

**Affiliations:** 1 Department of Computer Science and Engineering, National Sun Yat-sen University, Kaohsiung, Taiwan; 2 Artificial Intelligence Research and Promotion Center, National Sun Yat-sen University, Kaohsiung, Taiwan; 3 Department of Computer Science and Information Engineering, Providence University, Taichung, Taiwan; 4 Department of Computer Science and Information Engineering, Shu-Te University, Kaohsiung, Taiwan; 5 Department of Pediatrics, Kaohsiung Chang Gung Memorial Hospital, Kaohsiung, Taiwan; Bina Nusantara University, INDONESIA

## Abstract

Kawasaki Disease (KD) is a rare febrile illness affecting infants and young children, potentially leading to coronary artery complications and, in severe cases, mortality if untreated. However, KD is frequently misdiagnosed as a common fever in clinical settings, and the inherent data imbalance further complicates accurate prediction when using traditional machine learning and statistical methods. This paper introduces two advanced approaches to address these challenges, enhancing prediction accuracy and generalizability. The first approach proposes a stacking model termed the Disease Classifier (DC), specifically designed to recognize minority class samples within imbalanced datasets, thereby mitigating the bias commonly observed in traditional models toward the majority class. Secondly, we introduce a combined model, the Disease Classifier with CTGAN (CTGAN-DC), which integrates DC with Conditional Tabular Generative Adversarial Network (CTGAN) technology to improve data balance and predictive performance further. Utilizing CTGAN-based oversampling techniques, this model retains the original data characteristics of KD while expanding data diversity. This effectively balances positive and negative KD samples, significantly reducing model bias toward the majority class and enhancing both predictive accuracy and generalizability. Experimental evaluations indicate substantial performance gains, with the DC and CTGAN-DC models achieving notably higher predictive accuracy than individual machine learning models. Specifically, the DC model achieves sensitivity and specificity rates of 95%, while the CTGAN-DC model achieves 95% sensitivity and 97% specificity, demonstrating superior recognition capability. Furthermore, both models exhibit strong generalizability across diverse KD datasets, particularly the CTGAN-DC model, which surpasses the JAMA model with a 3% increase in sensitivity and a 95% improvement in generalization sensitivity and specificity, effectively resolving the model collapse issue observed in the JAMA model. In sum, the proposed DC and CTGAN-DC architectures demonstrate robust generalizability across multiple KD datasets from various healthcare institutions and significantly outperform other models, including XGBoost. These findings lay a solid foundation for advancing disease prediction in the context of imbalanced medical data.

## Introduction

Kawasaki Disease (KD), also known as Mucocutaneous Lymph Node Syndrome, primarily affects children under five years of age. This acute vasculitis is characterized by several key symptoms, including persistent fever lasting over five days, skin rashes, swollen and red lips, inflamed oral mucosa, non-purulent conjunctivitis, swollen and red hands and feet, and enlarged cervical lymph nodes [1, 2]. Without timely and appropriate treatment, these symptoms can progress to severe cardiovascular complications, including coronary artery dilation, myocardial infarction, and potentially fatal outcomes.

The diagnosis of KD is based on clinical criteria that include a fever for more than five days and at least four of the following five symptoms: mucocutaneous inflammation, including bilateral nonexudative conjunctivitis; mucositis; polymorphous skin rash; changes in extremities; and lymphadenopathy [3]. However, not all affected children exhibit four of these symptoms simultaneously, leading to potential misdiagnoses and errors in clinical judgment. Furthermore, the absence of a standard sequence for symptom onset often results in treatment delays, exacerbating the risk of serious complications [4].

Current KD prediction and diagnostic methods primarily fall into two categories: scoring systems [5–8] and machine learning approaches [9, 10]. While KD scoring systems assist in clinical diagnosis and evaluation, they present several limitations. These systems are highly dependent on physicians’ subjective judgments, which introduces variability and potentially inconsistent results across practitioners. Additionally, the specificity and sensitivity of scoring systems generally hover around 80%, leading to potential diagnostic errors, especially in patients with atypical symptoms.

In recent KD research, many machine learning models have employed individual algorithms such as XGBoost [11], AdaBoost [12], and CatBoost [13]. Consequently, these models often exhibit limitations in specificity, sensitivity, and generalizability. Training on data from a single hospital restricts their generalizability across diverse clinical environments [4]. Furthermore, these algorithms rely heavily on specific data features, making them vulnerable to dataset-specific characteristics and limiting their adaptability to new data. An individual algorithm may also fail to fully capture the complex clinical manifestations of KD, resulting in suboptimal diagnostic accuracy.

The low accuracy of KD machine learning models is largely due to the inherent challenges of KD as a classification problem, which requires sorting data into distinct classes [14]. Typically, machine learning classification tasks are structured as binary or multi-class problems. An imbalanced dataset often skews classifications, resulting in poor model performance, which is a significant issue that requires careful consideration [15]. Imbalanced data is common in binary classification problems, especially in medical contexts where datasets are divided into positive and negative instances. In such cases, positive cases represent diagnosed patients, while negative cases represent undiagnosed individuals. Clinical datasets usually contain far fewer positive cases than negative ones.

There are two main factors contributing to the imbalance in disease datasets. First, rare diseases are challenging to collect data for, as patients with these conditions are significantly fewer than those with more common diseases. Second, biases or errors during data collection [16], such as human error or unique cases, can also create imbalances. As a result, disease datasets in medical institutions often display imbalances, as observed in the KD dataset analyzed in this study.

This paper leverages CTGAN [17] to capture nonlinear data features, generating synthetic samples that closely mirror the true KD distribution. By modeling the conditional distribution of patient characteristics in the KD dataset, this approach addresses overfitting and data imbalance issues, producing samples highly representative of the original data. Consequently, CTGAN improves minority class recognition, substantially enhancing the model’s predictive accuracy. Thus, CTGAN is a reliable solution for handling KD’s data imbalance challenges, enabling machine learning models to achieve robust diagnostic performance in KD.

In summary, this paper’s innovation and contribution lie in using ensemble learning methods to integrate various machine learning algorithms, improving model accuracy and generalizability. Then, feature engineering addresses the KD dataset imbalance and optimizes the model by generating minority samples using CTGAN. This approach enhances reliability by validating synthetic samples via the CTGAN generator’s loss function and assessing them using the Jensen-Shannon Divergence (JSD) [18, 19] metric. Both validation techniques confirm that CTGAN outperforms traditional methods, such as random oversampling [20] and SMOTE [21], in generating reliable samples. Consequently, the effectiveness of machine learning models for diagnosing KD is enhanced.

The proposed model will be tested with KD data from two hospitals (Kaohsiung Chang Gung Memorial Hospital for model testing and Kaohsiung Medical University Chung-Ho Memorial Hospital for generalization validation). Experimental results show that the Disease Classifier (DC) of the proposed Stacking model and the CTGAN-DC model integrating DC and Conditional GAN have excellent generalization properties, especially CTGAN-DC, which shows excellent generalization properties. In different hospital settings, CTGAN-DC performs exceptionally well for the same type of disease, achieving both sensitivity and specificity of 95%. However, the model described in the JAMA journal’s [4] experiences Model Collapse during generalizability testing. Thus, the proposed model effectively meets the challenges posed by various clinical environments and improves the practical application value and generalizability of KD models.

The remainder of this paper is organized as follows: Related Works provides an overview of Kawasaki disease and discusses issues related to imbalanced disease datasets; Materials and Methods details the preprocessing of KD data and the proposed Ensemble Learning framework; Descriptive Statistical Analysis of Kawasaki Disease Data offers an overview of the dataset used in this study; Results presents the experimental findings, including the experimental setup and Ensemble Learning outcomes; finally, Conclusion summarizes the research and suggests directions for future studies.

## Related works

Kawasaki Disease (KD) is a common vasculitis in early childhood that may lead to acquired heart disease [3, 22]. Most cases related to KD occur in patients with giant coronary artery aneurysms. If KD patients receive intravenous immunoglobulin (IVIG) treatment within the first ten days of onset, the incidence of coronary artery aneurysms decreases from 25% to 4%. Therefore, early recognition of KD and prompt IVIG treatment is crucial in reducing severe complications and preventing childhood-related mortality.

The main method for treating KD is Intravenous Immunoglobulin (IVIG). [23] proposes a multi-stage approach combining co-clustering and machine learning to predict IVIG resistance in KD patients. The goal is to enhance and optimize the treatment methods for patients who exhibit resistance to IVIG. Additionally, other related studies are addressing IVIG resistance in KD [24, 25]. In recent years, emerging studies [26, 27] have been on methods for identifying KD patients and febrile children. However, these methods are still at a distance from practical clinical application.

Current research methodologies in the literature are primarily categorized into scoring systems and machine learning techniques. Existing studies indicate that machine learning significantly outperforms scoring systems in identifying KD patients and distinguishing febrile children. However, a key challenge in applying machine learning models to KD diagnosis is data imbalance. KD datasets are typically highly imbalanced, with significantly fewer positive KD cases compared to negative febrile cases, which can bias model predictions. To address this issue, prior studies have explored techniques such as random oversampling and SMOTE to balance datasets, thereby enhancing model prediction performance. This highlights the importance of dataset balancing in improving model sensitivity and specificity. Consequently, this paper will review the literature on machine learning applications in KD diagnosis and examine findings related to handling imbalanced disease datasets [28].

### Machine learning techniques for Kawasaki Disease diagnosis

M. Zhang et al. [29] utilized a gene expression dataset and machine learning techniques to develop a diagnostic model for distinguishing KD patients. They analyzed data from the Gene Expression Omnibus (GEO) using Random Forest (RF) and Artificial Neural Network (ANN). The model demonstrated excellent performance in differentiating KD patients, convalescent patients, and healthy individuals, with AUC values between 0.945 and 1. However, the study did not provide sensitivity and specificity values. They highlighted limitations in data processing and classification criteria, indicating the need for further validation and optimization in future research.

JY. Lam et al. [30] introduced KIDMATCH, a machine learning algorithm to distinguish Multisystem Inflammatory Syndrome in Children (MIS-C), KD, and other similar acute febrile illnesses. The study utilized patient age, the five classic clinical symptoms of KD, and 17 laboratory test values. The algorithm was trained through a two-stage deep learning model. During internal validation, the second stage achieved an AUC of 96.0%, with a sensitivity of 95% and specificity of 87.6%. The study highlighted potential challenges the model might encounter with non-standardized laboratory data, recommending broader validation across various regions and populations. Additionally, the KIDMATCH model requires better integration into clinical workflows, and future research should aim to enhance the model’s generalizability and practical utility.

C. Li et al. [31] developed a machine learning model to differentiate between KD and sepsis using clinical data. The study involved 299 KD patients and 309 sepsis patients, collecting data on age, gender, height, weight, BMI, and 33 routine blood parameters. Key variables were identified using the LASSO method and a support vector machine model. The model achieved a sensitivity of 86.8% and a specificity of 84.6%. However, the study’s limitations include being conducted at a single center and the lack of external validation. Future research should include randomized controlled trials and external validation to reinforce the findings.

M.A. Portman et al. [32] applied artificial intelligence to blood tests to diagnose KD. They used the Least Absolute Shrinkage and Selection Operator (LASSO) and Minimum Angle Regression to identify 3 key biomarkers from 11 candidates: C-reactive protein, N-terminal pro-brain natriuretic peptide, and thyroid hormone uptake. The model achieved an AUC of 0.94 in 10-fold cross-validation, with a sensitivity of 94% and specificity of 91%. However, the study had limitations, including a small sample size and a lack of external validation. Future research should include larger samples and rigorous control group designs to validate the model’s effectiveness and applicability. Moreover, the machine learning model may require further adjustments to improve its diagnostic capability for KD, especially regarding its generalizability across different populations and clinical conditions.

Y. Duan et al. [10] developed a machine learning model to predict Kawasaki Disease (KD) in children from Chongqing, China, using seven different algorithms. Key predictive features, including platelet distribution width (PDW), erythrocyte sedimentation rate (ESR), and total protein (TP), were identified. The EBM model demonstrated superior performance compared to black-box models (e.g., LGBM and AdaBoost) in terms of discrimination, calibration, and interpretability, offering clinically valuable insights. The interpretability of black-box models depends on post-hoc techniques such as SHAP and LIME, which can introduce additional uncertainty. While data sourced from a single hospital may limit the generalizability of the findings, this study underscores the importance of intrinsic interpretability in clinical applications and recommends further validation to enhance broader applicability.

C.-M Tasi et al. [4] used machine learning to identify KD among febrile children in emergency rooms. They analyzed data from 74,641 febrile children across four hospitals, using the XGBoost algorithm to build the predictive model. The study suggests that objective laboratory test results can be effective parameters for predicting KD. However, since the dataset contains relatively few KD cases and primarily features general febrile illnesses, the model’s applicability, generalizability, and universality might be limited. Future research should validate the model’s effectiveness in more diverse and larger populations. The model’s design needed to address generalizability, potentially limiting its use in different medical settings.

### Techniques for processing imbalanced datasets in disease diagnosis

Previous machine learning-based studies on KD prediction reveal that data imbalance has become a critical challenge in applying machine learning models to medical predictions and diagnoses. For datasets involving rare diseases like KD, it is common for the minority class (KD cases) to be significantly outnumbered by the majority class (fever cases). This imbalance leads models to overlook the minority class, which significantly reduces diagnostic accuracy for rare diseases [33]. In the case of KD, a rare pediatric condition, the data imbalance issue is especially pronounced as fever cases far outnumber KD cases in clinical settings. Consequently, prediction models tend to produce a higher false-negative rate when diagnosing KD [34].

Common methods include oversampling, undersampling, and SMOTE algorithms to address the issue of imbalance in medical datasets. These techniques are widely used in handling imbalanced data issues for rare diseases, such as in the KD dataset. Oversampling, which increases the proportion of minority class samples by generating new examples, is particularly suited for imbalanced medical datasets as it helps avoid the data loss that undersampling might cause. However, oversampling can introduce data noise and even lead to model overfitting if the generated samples do not fully reflect the actual distribution, making it challenging for the model to perform well with real-world data. For instance, Random Over Sampling (ROS) balances the dataset by randomly duplicating minority class samples, providing a simple yet effective solution. Meanwhile, Adaptive Synthetic Sampling (ADASYN) assigns different weights based on the difficulty of learning minority samples, generating synthetic data that focuses on harder-to-learn samples [34, 35].

Various variations of Synthetic Minority Oversampling Techniques (SMOTE) have been extensively applied in the literature. SMOTE-NC, for instance, effectively balances continuous data, while Borderline-SMOTE focuses on oversampling boundary samples to enhance model sensitivity to edge data. SVM SMOTE leverages support vector machines to achieve boundary-focused oversampling, and KMeans SMOTE integrates k-means clustering better to represent minority classes [35, 36].

For imbalanced data generation, widely used techniques such as GReaT [37], CTGAN [17, 40], TVAE [17, 41], and CopulaGAN [38] demonstrate robust capabilities in handling heterogeneous data and generating high-quality synthetic samples. GReaT, for example, employs a large language model (LLM) to generate text-based data while preserving semantics and context, making it especially suitable for heterogeneous datasets. However, GReaT’s complexity makes it most appropriate for applications demanding high contextual consistency.

CTGAN provides significant advantages for addressing data imbalance, especially with datasets containing non-Gaussian distributions or diverse categorical variables. It effectively overcomes the curse of dimensionality associated with high-cardinality categorical variables, excelling at generating conditionally distributed data. These capabilities make CTGAN particularly suitable for Kawasaki Disease (KD) applications, which require handling heterogeneous medical data. Given the multidimensional nature of KD diagnostics, including blood and urine test data, CTGAN can capture complex feature distributions to create synthetic data for model training, thereby enhancing the generalization performance of diagnostic models. Consequently, CTGAN outperforms other methods like GReaT, TVAE, and CopulaGAN in medical diagnostic applications.

Undersampling is a technique used to balance datasets by reducing the number of samples in the majority class, although this approach can lead to the loss of significant data [35]. However, in medical datasets, excessive undersampling may overly reduce majority class data, potentially compromising the model’s predictive accuracy. Some commonly employed undersampling techniques include Random Undersampling (RUS), which randomly removes majority class samples to decrease data volume [35]; All k-nearest Neighbors (All k-NN), which eliminates majority class samples based on k-nearest neighbor classification [34]; Cluster Centers (CC), where majority class samples are replaced by cluster center points [39]; and Edited Nearest Neighbors (ENN), which removes misclassified samples according to nearest neighbor classification results [36].

In handling rare diseases like Kawasaki Disease (KD), both oversampling and undersampling techniques offer unique value but come with distinct drawbacks. Oversampling increases the proportion of minority classes by generating synthetic data, which can enhance model balance. However, synthetic data may introduce noise, especially if the original dataset includes anomalies or unclear boundaries, potentially exacerbating these issues. Additionally, oversampling can lead to model overfitting, particularly when the minority class size is very small; the synthetic samples may fail to fully capture the diversity within KD cases, which can reduce the model’s generalizability and accuracy in clinical applications. Conversely, due to the disease’s rarity, undersampling presents challenges in KD applications. Excessively reducing the majority class (negative samples) could lead the model to lose critical information necessary for distinguishing KD, increasing the risk of misdiagnosis. Furthermore, undersampling may result in the model overfitting on the limited minority samples, impairing its generalization capability.

According to the analysis above, the literature review suggests a range of diagnostic methods and data imbalance issues in KD prediction, emphasizing the use of machine learning techniques for patient identification. Most studies indicate that machine learning models significantly outperform traditional scoring systems in distinguishing KD patients from other febrile children. However, factors such as data imbalance have led to relatively low sensitivity and specificity in models like Random Forest (RF), Artificial Neural Networks (ANN), the KIDMATCH deep learning model, LASSO support vector machine, and KD-CNN. Furthermore, these approaches lack cross-hospital validation, highlighting substantial limitations in generalizability.

This paper proposes two novel ensemble learning methods that combine multiple machine learning algorithms to enhance model stability and generalizability. Specifically, the Disease Classifier (DC) and CTGAN-DC models employ CTGAN data augmentation, feature engineering, and model optimization on imbalanced Kawasaki Disease (KD) clinical data, leading to significant improvements in diagnostic accuracy. These methods utilize clinical data from Kaohsiung Chang Gung Memorial Hospital and Kaohsiung Medical University Chung-Ho Memorial Hospital for testing and validation to assess model usability and generalizability. Experimental results show that the DC and CTGAN-DC models achieve high generalizability. On the KD test set from Kaohsiung Chang Gung Memorial Hospital, the DC model achieved a sensitivity of 95% and a specificity of 94%, while the CTGAN-DC model achieved a sensitivity of 95% and a specificity of 97%, outperforming XGBoost and other models in the literature. In the validation set from Kaohsiung Medical University Chung-Ho Memorial Hospital, the CTGAN-DC model achieved both a sensitivity and specificity of 95%, surpassing the XGBoost model referenced in JAMA [4], which encountered model collapse issues during validation testing.

## Materials and methods

The primary objective of this paper is to address the persistent issue of data imbalance in medical disease datasets, exemplified by Kawasaki Disease (KD). Previous research often employs synthetic data generation techniques to alleviate this imbalance, leveraging various advanced methods such as GReaT (Generation of Realistic Tabular Data) [37], CTGAN (Conditional Tabular GAN) [17, 40], TVAE (Tabular Variational Autoencoder) [17, 41], and CopulaGAN [38]. These techniques generate synthetic samples for the minority class to enhance model prediction performance. This study specifically applies CTGAN for data augmentation, aiming to significantly enhance the accuracy and generalizability of the KD prediction model.

The KD dataset presents an extreme imbalance in its binary classification, with the positive class representing KD patients and the negative class consisting of cases with general fever (body temperature exceeding 37.5°*C*). Despite attempts in previous studies to distinguish KD from general fever cases, several substantial challenges limit these models’ clinical applicability.

Prior research identifies three primary issues: first, a lack of supplementary validation data limits the verification of the selected features’ effectiveness. Second, many models struggle to generalize effectively across different datasets, resulting in limited adaptability to various medical institutions. Lastly, while achieving high true positive rates, many also show elevated false positive rates, which undermines these models’ reliability in clinical applications. In response, this study introduces two architectures—Disease Classifier (DC) and Disease Classifier with CTGAN (CTGAN-DC)—to address data imbalance and enhance model performance.

The DC and CTGAN-DC architectures incorporate ensemble learning, oversampling, and stacking techniques. The DC architecture focuses on improving minority class representation, while CTGAN-DC integrates CTGAN technology within the DC framework to augment the diversity of KD data samples while retaining their intrinsic characteristics [17]. By tackling data imbalance and improving generalizability, these architectures help mitigate limitations noted in previous studies, leading to improved accuracy and robustness in KD prediction models. The following sections provide a detailed description of the proposed architectures and their respective functionalities.

The research framework employs real-world imbalanced KD data from Kaohsiung Chang Gung Memorial Hospital and Kaohsiung Medical University Chung-Ho Memorial Hospital. The Chang Gung Medical Foundation Institutional Review Board approved this study (IRB numbers 202202165B0 and 202202165B0C501). The IRB approved a waiver of participants’ consent, as all collected data were de-identified, ensuring that team members could not identify individual participant information. The data for this study were accessed and collected following IRB approval, starting from February 7, 2023. The data collection protocols and related documentation were also reviewed and approved.

The medical data encompass various types of information, making the feature selection process crucial. After appropriate data cleaning and transformation, these data can serve as training, test, and validation data for the model. The KD dataset *D* = *D*_1_∪*D*_2_ is divided into a training set *D*_1_ and a test set *D*_2_. Each (*X*_*i*_, *y*_*i*_) represents the *i*-th sample, where *X*_*i*_ is the feature vector and *y*_*i*_ is the label (0 or 1). The index *i* ranges from 1 to *I*, with *I* being the number of samples in the training set. The label *y*_*i*_ is binary, meaning *y*_*i*_ ∈ [0, 1].

$$\begin{array}{c} D_{1}=\left(X_{i},y_{i})|i=1,2,3,\ldots ,I,y_{i}\in [0,1\right] \end{array}$$ (1)

Each (*X*_*j*_, *y*_*j*_) represents the *j*-th sample, where *X*_*j*_ is the feature vector and *y*_*j*_ is the label (0 or 1). The index *j* ranges from 1 to *J*, with *J* being the number of samples in the training set. The label *y*_*j*_ is binary, meaning *y*_*j*_ ∈ [0, 1].

$$\begin{array}{c} D_{2}=\left(X_{j},y_{j})|j=1,2,3,\ldots ,J,y_{j}\in [0,1\right] \end{array}$$ (2)

In an imbalanced binary classification problem, we can represent *D* as *D* = *D*_*KD*_*major*_ ∪ *D*_*KD*_*minor*_. The proportions of *D* are significantly disparate:

$$\begin{array}{c} \left|D_{KD\_major}|\gg |D_{KD\_minor}\right| \end{array}$$ (3)

Therefore, predicting minority samples is more challenging for the model. In this study, we developed two ensemble learning methods, DC and CTCAN-DC. Our ultimate goal is to enhance the clinical prediction accuracy and generalization of KD in imbalanced binary classification problems using Ensemble Learning.

### Data preprocessing

With the rapid advancements in machine learning, the significance of data quality for models has become increasingly apparent. Data quality and feature selection directly impact a model’s potential performance. Therefore, post-collection data processing is essential. This study utilizes medical records from Kaohsiung Chang Gung Memorial Hospital (2010-2019) spanning ten years and from Kaohsiung Medical University Chung-Ho Memorial Hospital (2012-2020), encompassing nine years. As Kawasaki Disease (KD) diagnosis requires a persistent fever lasting over five days, which predominantly affects young children, we selected febrile children (with a body temperature above 37.5°C) as the control group. The age range for both datasets is restricted to children aged 0 to 5 years (not including five years). For this research, we identified 22 features spanning basic patient demographics, blood tests, and urine tests, as shown in Table 1.

**Table 1:** The features of the KD datasets.

	Features
**Demographic characteristics**	Age
	Sex
	Date of visit
**Blood**	RBC (Red Blood Cell)
	WBC (White Blood Cell)
	Hemoglobin
	Hematocrit
	MCH (Mean Corpuscular Hemoglobin)
	MCHC (Mean Corpuscular Hemoglobin Concentration)
	RDW (Red Blood Cell Volume Distribution Width)
	Platelets
	Neutrophils-segments
	Neutrophils-bands
	Lymphocyte
	Monocyte
	Eosinophils
	Basophils
	AST (Aspartate Transaminase)
	ALT (Alanine Transaminase)
	CRP (C-Reactive Protein)
**Urine**	WBC count in urine
	Pyuria

This paper refers to the current literature that has performed the best in predicting KD based on machine learning [4] for feature selection. Our dataset, however, is notably distinct in its feature selection and acquisition, incorporating more critical features pertinent to KD analysis. For example, the model in [29] depends on gene expression data, such as those available from GEO, which entails high costs and specialized expertise. In contrast, this study utilizes routine, widely accessible clinical test data—including red blood cells, white blood cells, and C-reactive protein—making it more practical for real-world applications.

In comparison, another model, KIDMATCH [30], incorporates five primary clinical symptoms and several laboratory tests. However, symptom analysis in KIDMATCH is subject to subjective judgment, resulting in high data variability. This study, instead, emphasizes standardized data, aiming to improve stability. While much of the existing literature is limited to a narrow range of blood biomarkers for KD diagnosis, our research includes C-reactive protein alongside additional indicators like neutrophil differentiation, RDW, eosinophils, and basophils, offering a more comprehensive view of KD pathology. Furthermore, it incorporates liver function indicators, such as ALT and AST, which may be valuable for KD diagnosis, thereby demonstrating its practical utility in clinical settings.

In clinical practice, medical datasets frequently present issues such as incomplete or missing data, outliers, and noise. These challenges primarily stem from the manual recording processes used in hospital data collection, which make the data vulnerable to missing values. Additionally, the feature set in the KD dataset we are using includes two types of examinations—blood and urine. However, not all patients undergo both tests, particularly the urine test, which demands substantial medical resources. These factors substantially contribute to the occurrence of missing values.

To solve the issues above, we follow the professional advice of clinicians and implement two main steps. The first step is data cleaning. Initially, we select febrile children from the union of datasets *D* as indicated by (1) and (2). We then exclude cases with three or more missing test items. Therefore, if a sample *i* in *X*_*i*_ has three or more missing values (*M*_*i*_ ≥ 3), we remove that sample, as shown in (4) and (5). $D_{1}^{\prime }$ is the training set after removing samples with more than three missing values, while $D_{2}^{\prime }$ is the test set after removing such samples.

$$\begin{array}{c} D_{1}^{\prime }=\left\{(X_{i},y_{i})|(X_{i},y_{i})\in D_{1},M_{i}<3\right\} \end{array}$$ (4)

$$\begin{array}{c} D_{2}^{\prime }=\left\{(X_{j},y_{j})|(X_{j},y_{j})\in D_{2},M_{j}<3\right\} \end{array}$$ (5)

We fill in the remaining missing values using the mean of the corresponding test items. Let *μ*_*k*,*D*1_ and *μ*_*k*,*D*2_ represent the mean of the *k*-th test item in datasets $D_{1}^{\prime }$ and $D_{2}^{\prime }$, respectively. We impute each missing value *X*_*i*,*k*_ (i.e., the *k*-th test item of sample *i*) according to (6).

$$\begin{array}{c} X_{i,k}=\{\begin{array}{cc} X_{i,k} & \text{if}\;X_{i,k}\;\text{is}\;\text{not}\;\text{missing}\\ \mu_{k,D1} & \text{if}\;X_{i,k}\;\text{is}\;\text{missing}\;\text{and}\;\text{belongs}\;\text{to}\;D1^{\prime }\\ \mu_{k,D2} & \text{if}\;X_{i,k}\;\text{is}\;\text{missing}\;\text{and}\;\text{belongs}\;\text{to}\;D2^{\prime } \end{array} \end{array}$$ (6)

The second step is to ensure data independence. Patients with multiple clinic visits due to persistent fever may have multiple blood and urine tests. Thus, we use the most recent test before diagnosis as the result. If the interval between visits for the same patient is less than two weeks, we exclude those records, as shown in (7) and (8). The consecutive patient visit records are denoted as *R*_*i*_.

$$\begin{array}{c} D_{1}^{\prime \prime }=\{(X_{i},y_{i})|(X_{i},y_{i})\in D_{1}^{\prime },\;R_{i}\geq 14\;days \end{array}$$ (7)

$$\begin{array}{c} D_{2}^{\prime \prime }=\{(X_{j},y_{j})|(X_{j},y_{j})\in D_{2}^{\prime },\;R_{i}\geq 14\;days \end{array}$$ (8)

In this way, $D_{1}^{\prime \prime }$ and $D_{2}^{\prime \prime }$ ensure the independence of the data and prevent the risk of overfitting during model training caused by closely spaced visits. Apart from the necessary preprocessing steps mentioned above, we decided not to apply additional processing or feature engineering to the remaining feature fields. This design has two main advantages: data integrity and medical interpretability. Keeping the original dataset intact helps maintain data integrity, ensuring that important information is not lost due to excessive processing. Moreover, this approach reduces the computational cost of preprocessing and enhances processing efficiency. Additionally, preserving the original data characteristics aids medical interpretability, making the research results more straightforward to understand and apply in clinical practice.

### Classification model

Ensemble Learning combines multiple machine learning models for more accurate and stable predictions. Its strength lies in integrating the advantages of various models and utilizing their diversity and complementarity to enhance overall generalization ability. Using stacking, different algorithms or parameters are combined to capture distinct features and patterns, fitting the data better. Moreover, it improves the model’s generalization capacity and reduces the risk of overfitting. This study designs DC and CTGAN-DC frameworks based on Ensemble Learning, oversampling, and stacking principles.

#### Disease Classifier (DC)

The DC model architecture employs a two-stage training process. In the first stage, we use K-fold cross-validation to train the first layer of models. We start by splitting the training data $D_{1}^{\prime \prime }$ into five parts, as shown in (9).

$$\begin{array}{c} D_{1}^{\prime \prime }=D_{1,1}\cup D_{1,2}\cup D_{1,3}\cup D_{1,4}\cup D_{1,5} \end{array}$$ (9)

In (10), we use four of the five parts as the training set to train each classifier model in the first layer, denoted as $D_{1,train}^{\left(k\right)}$. The remaining part is used as the validation set, denoted as $D_{1,val}^{\left(k\right)}$. Here, $D_{1,train}^{\left(k\right)}$ represents the training dataset in the *k*-th iteration, which consists of $D_{1}^{\prime \prime }$ excluding the *k*-th part. $D_{1,val}^{\left(k\right)}$ represents the validation dataset in the *k*-th iteration, which is the *k*-th part of $D_{1}^{\prime \prime }$.

$$\begin{array}{c} D_{1,\text{train}}^{\left(k\right)}=D_{1}^{\prime \prime }\backslash D_{1,k}\\ D_{1,\text{val}}^{\left(k\right)}=D_{1,k} \end{array}$$ (10)

In each iteration, we train the classifier model with the training set $D_{1,\text{train}}^{\left(k\right)}$ and test the trained model with the validation set $D_{1,\text{val}}^{\left(k\right)}$. The predicted classification probabilities are used as features. After repeating this process five times, we obtain five probability features. These features are then combined to form the final output of the first layer.

Balancing model performance and computational efficiency is essential when selecting the number of base classifiers. An appropriate number of base classifiers can provide diversity, enhancing the model’s generalization ability and stability. However, selecting too many base classifiers may not significantly improve performance and will increase computational and storage costs. This study selects three machine learning classification algorithms as the base classifiers for the first layer of training: XGBoost, AdaBoost, and CatBoost. The set of base classifiers is denoted as B:

$$\begin{array}{c} B=\{XGBoost,AdaBoost,CatBoost\} \end{array}$$ (11)

In (12), the index of the base classifier *b* is defined as b∈B, where the set of base classifiers B includes multiple different classifiers. During the cross-validation process, *k* represents the *k*-th iteration. This study conducts cross-validation with *K* = 5 iterations. In each iteration, a different dataset $D_{1,\text{train}}^{\left(k\right)}$ is used, with part of it serving as the validation set $D_{1,\text{val}}^{\left(k\right)}$.

The term ${{\hat{y}}_{i}}^{\left(k,b\right)}$ represents the predicted probability produced by the *b*-th base classifier for the *i*-th validation sample during the *k*-th cross-validation. This value indicates the probability that the sample *X*_*i*,val_ is classified as a specific category, such as negative or positive. The function $f_{b}\left(X_{i,\text{val}}|D_{1,\text{train}}^{\left(k\right)}\right)$ is the prediction function of the *b*-th base classifier, trained on the training set $D_{1,\text{train}}^{\left(k\right)}$, and outputs the predicted probability that the sample *X*_*i*,val_ belongs to a specific category. *X*_*i*,val_ represents the feature vector of the *i*-th sample in the validation dataset.

$$\begin{array}{c} {{\hat{y}}_{i}}^{\left(k,b\right)}=f_{b}(X_{i,\text{val}}|D_{1,\text{train}}^{\left(k\right)}),\;X_{i,\text{val}}\in D_{1,\text{val}}^{\left(k\right)},\;b\in B \end{array}$$ (12)

This process will be repeated five times, using *k* − 1 parts as the training set. Ultimately, this will produce five sets of predicted probability features. These features will be aggregated using (13) to form the final output of the first layer, ${\hat{Y}}_{1}$.

$$\begin{array}{c} \hat{Y_{1}}=\overset{5}{\underset{k=1}{⋃}}\underset{b\in B}{⋃}\{{\hat{y_{i}}}^{\left(k,b\right)}\} \end{array}$$ (13)

The three algorithms in B have unique characteristics and advantages. Combining their prediction results can achieve a more comprehensive and accurate model performance. Additionally, we selected these three algorithms to balance performance and efficiency, ensuring good performance within resource and time constraints.

In the second stage, we use the prediction results $\hat{Y_{1}}$ from the previous three base classifiers as features to create a new feature matrix **Z**, as shown in (14). **Z** will then serve as the input for the final meta-model. *N* is the total number of samples in the dataset.

$$\begin{array}{c} Z=[\begin{array}{ccc} {\hat{y}}_{1}^{\left(1,\text{XGBoost}\right)} & {\hat{y}}_{1}^{\left(1,\text{AdaBoost}\right)} & {\hat{y}}_{1}^{\left(1,\text{CatBoost}\right)}\\ {\hat{y}}_{2}^{\left(2,\text{XGBoost}\right)} & {\hat{y}}_{2}^{\left(2,\text{AdaBoost}\right)} & {\hat{y}}_{2}^{\left(2,\text{CatBoost}\right)}\\ \vdots & \vdots & \vdots \\ {\hat{y}}_{N}^{\left(5,\text{XGBoost}\right)} & {\hat{y}}_{N}^{\left(5,\text{AdaBoost}\right)} & {\hat{y}}_{N}^{\left(5,\text{CatBoost}\right)} \end{array}] \end{array}$$ (14)

Since this study aims to solve the problem of imbalanced binary classification, the final meta-model is trained using Logistic Regression, as shown in (15). We balance the importance between the two classes by adjusting the weights. For sample *i*, the true label is *y*_*i*_ (1 indicates positive, 0 indicates negative), and the probability of predicting it as positive is denoted as ${\hat{y}}_{DC,i}$.

$$\begin{array}{c} l(w)=\sum_{i=1}^{N}[y_{i}\text{log}({\hat{y}}_{DC,i})+(1-y_{i})\text{log}(1-{\hat{y}}_{DC,i})] \end{array}$$ (15)

To ensure the model focuses more on minority class samples and improves accuracy for the minority class, we can introduce weights *α*_*i*_ to the log-likelihood function, as shown in (16). Here, *α*_*i*_ is the weight of class *i*, assigning higher weights to the minority class samples.

$$\begin{array}{c} l(w)=\sum_{i=1}^{N}\alpha_{i}[y_{i}\text{log}({\hat{y}}_{DC,i})+(1-y_{i})\text{log}(1-{\hat{y}}_{DC,i})] \end{array}$$ (16)

In (17), the Logistic Regression model calculates the predicted probability ${\hat{y}}_{DC,i}$ that the *i*-th sample is classified as positive. Here, *σ* represents the sigmoid function, *w* is the model’s weight vector, and *z*_*i*_ is the *i*-th row of the feature matrix *Z*, which is the predicted probability feature vector of the *i*-th sample.

$$\begin{array}{c} {\hat{y}}_{DC,i}=\sigma (w^{⊤}z_{i})=\frac{1}{1+\text{exp}(-w^{⊤}z_{i})} \end{array}$$ (17)

**Algorithm 1 Pseudo-code of Disease Classifier (DC)**.

1: **Input:** KD dataset *D* = *D*_1_∪*D*_2_, containing features *X*_*i*_ and labels *y*_*i*_

2: **Initialization:** K-fold cross-validation (*K* = 5), base classifiers (XGBoost, AdaBoost, CatBoost)

3: **Output:** Predicted labels for the test set

4: ***func*** TrainDCModel(Dataset *D*_1_)

5:  Split *D*_1_ into 5 folds: *D*_1,1_, *D*_1,2_, *D*_1,3_, *D*_1,4_, *D*_1,5_

6:  Initialize base classifiers: *B* = {XGBoost, AdaBoost, CatBoost}

7:  **for** *k* = 1 to 5 **do**

8:    Train set $D_{1,\text{train}}^{\left(k\right)}=D_{1}\backslash D_{1,k}$

9:    Validation set $D_{1,\text{val}}^{\left(k\right)}=D_{1,k}$

10:   **foreach** base classifier *b* in *B* **do**

11:    Train classifier *b* using $D_{1,\text{train}}^{\left(k\right)}$

12:    Predict on validation set $D_{1,\text{val}}^{\left(k\right)}$ and store predicted probabilities

13:    Store probabilities ${\hat{y}}_{i}^{\left(k,b\right)}$ for each sample in validation set

14:   **end for**

15:  **end for**

16:  Combine probabilities from each fold to form final layer output ${\hat{Y}}_{1}$

17:  Train meta-model using Logistic Regression with ${\hat{Y}}_{1}$ as features

18:  **return** Trained DC model

19: **end func**

Another reason for choosing Logistic Regression, as previously mentioned, is that the first layer of base classifiers involves complex computations. Therefore, in the second layer, we use a simple linear combination to capture the relationships among predicted probability features. The entire Stacking Ensemble Learning Disease Classifier (Stacking DC model) is evaluated using the test dataset $D_{2}^{\prime \prime }$, and its performance surpasses that of all existing Kawasaki Disease research methods, as shown in Algorithm 1.

#### Disease Classifier with CTGAN (CTGAN-DC)

**Fig 1:**
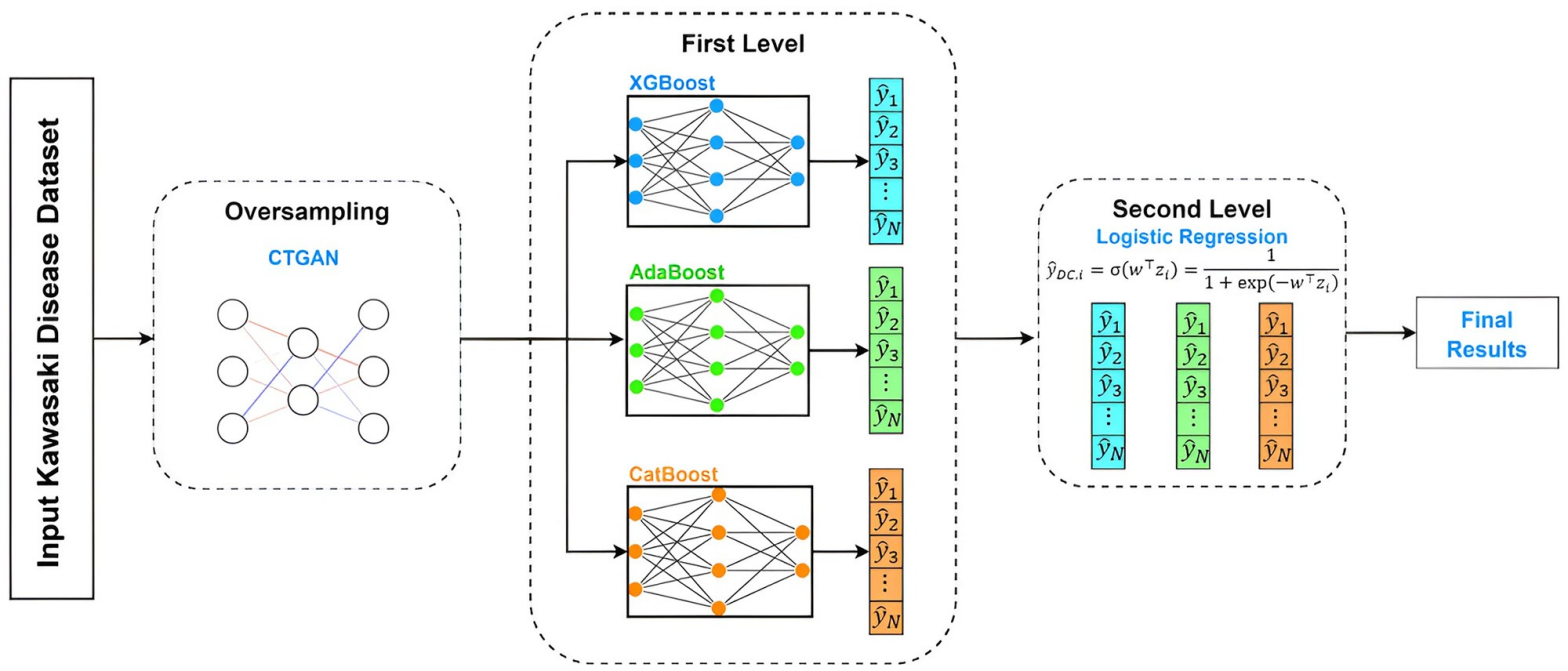
Disease Classifier with CTGAN (CTGAN-DC) method. The CTGAN-DC is based on DC for Oversampling (CTGAN), Stacking, and Ensemble Learning architecture. Through the Oversampling concept, CTGAN generates KD minority class data and uses the Stacking Model to divide it into First Level and Second Level training models. Experimental results prove that it effectively improves KD prediction sensitivity and specificity. At the same time, this study found that architecture has generalization performance.

The CTGAN-DC model is an improvement based on the DC model, using the original imbalanced medical data as the training dataset *D*′ in the initial training process. However, the design concept of CTGAN-DC aims to retain the raw data while increasing its diversity through oversampling. Therefore, training data with different positive and negative ratios are input into the model to explore whether better prediction results can be achieved, as shown in Fig 1.

$$\begin{array}{c} D^{\prime }=D_{1}^{\prime \prime }\cup D_{2}^{\prime \prime } \end{array}$$ (18)

The overall method consists of three main steps. First, to balance data with different positive and negative ratios for training, we introduce the CTGAN method to generate synthetic data using Eq (19). Here, *h*_*j*_ represents noise vectors randomly sampled from a multivariate normal distribution, MVN(0, *I*), where the mean is 0, and the covariance matrix is the identity matrix *I*. The index *j* indicates each sample in the batch, ranging from 1 to *m*, where *m* is the batch size, i.e., the number of samples processed in each training iteration. Thus, *j* ranges from 1 ≤ *j* ≤ *m*, meaning there are *m* samples involved in each training iteration.

$$\begin{array}{c} h_{j}∼\text{MVN}(0,I)\;\;\;\text{for}\;1\leq j\leq m \end{array}$$ (19)

Using the conditional vector cond_*j*_, we generate the synthetic data sample ${\tilde{r}}^{j}$. The sample ${\tilde{r}}^{j}$ is produced by the generator function *G* based on *h*_*j*_ and cond_*j*_, i.e., ${\tilde{r}}^{j}=G\left(h_{j},{\text{cond}}_{j}\right)$. Specifically, ${\tilde{r}}^{j}$ represents the *j*-th generated synthetic data sample. The function *G* combines the random noise vector *h*_*j*_ and the conditional vector cond_*j*_ to generate the synthetic data sample:

$$\begin{array}{c} {\tilde{r}}^{j}=G(h_{j},{\text{cond}}_{j})\;\;\;\text{for}\;1\leq j\leq m \end{array}$$ (20)

Next, we sample a real data sample *r*_*j*_ from the dataset *D*′ that meets the condition cond_*j*_:

$$\begin{array}{c} r_{j}∼U(D^{\prime }|{\text{cond}}_{j})\;\;\;\text{for}\;1\leq j\leq m \end{array}$$ (21)

In (22), (23), and (24) will be combined into batches, with *p*_ac_ samples grouped into each batch. Thus, the synthetic data batch ${\tilde{r}}^{k}\left(p_{\text{ac}}\right)$, the real data batch *r*^*k*^(*p*_ac_), and the conditional vector batch cond^*k*^(*p*_ac_) are formed:

$$\begin{array}{c} {\text{cond}}^{k}(p_{\text{ac}})={\text{cond}}^{k\times p_{\text{ac}}+1}⊕\cdots ⊕{\text{cond}}^{k\times p_{\text{ac}}+p_{\text{ac}}}\\ \;\;\;\text{for}\;1\leq k\leq \frac{m}{p_{\text{ac}}} \end{array}$$ (22)

$$\begin{array}{c} {\tilde{r}}^{k}(p_{\text{ac}})={\tilde{r}}^{k\times p_{\text{ac}}+1}⊕\ldots ⊕{\tilde{r}}^{k\times p_{\text{ac}}+p_{\text{ac}}}\\ \;\;\;\text{for}\;1\leq k\leq \frac{m}{p_{\text{ac}}} \end{array}$$ (23)

$$\begin{array}{c} r^{k}(p_{\text{ac}})=r^{k\times p_{\text{ac}}+1}⊕\ldots ⊕r^{k\times p_{\text{ac}}+p_{\text{ac}}}\\ \;\;\;\text{for}\;1\leq k\leq \frac{m}{p_{\text{ac}}} \end{array}$$ (24)

Calculate the gradient descent of the loss function *L*_*GP*_:

$$\begin{array}{c} L_{\text{GP}}=\frac{1}{m/\text{pac}}\sum_{k=1}^{m/\text{pac}}{\left(\left|\right|\nabla_{{{\tilde{r}}_{k}}^{\left(\text{pac}\right)}}\text{Critic}({\tilde{r}}^{\left(\text{pac}\right)},{\text{cond}}_{k}^{\left(\text{pac}\right)}){\left|\right|}_{2}-1\right)}^{2} \end{array}$$ (25)

Update the discriminator parameters Φ_*C*_:

$$\begin{array}{c} \Phi_{C}\leftarrow \Phi_{C}-\lambda \cdot \text{Adam}(\nabla_{\Phi_{C}}(L_{C}+\gamma L_{\text{GP}})) \end{array}$$ (26)

Calculate the generator loss *L*_*G*_:

$$\begin{array}{cc} L_{G}= & -\frac{1}{m/\text{pac}}\sum_{k=1}^{m/\text{pac}}\text{Critic}({{\hat{r}}_{k}}^{\left(pac\right)},{\text{cond}}_{k}^{\left(\text{pac}\right)})\\ \; & +\frac{1}{m}\sum_{j=1}^{m}\text{CrossEntropy}(d_{i^{✱},j},m_{i^{✱}}) \end{array}$$ (27)

Update the generator parameters Φ_*G*_:

$$\begin{array}{c} \Phi_{G}\leftarrow \Phi_{G}-\lambda \cdot \text{Adam}(\nabla_{\Phi_{G}}L_{G}) \end{array}$$ (28)

CTGAN is an advanced synthetic data generation technique that can simulate and generate synthetic data conforming to the raw data distribution. Through CTGAN oversampling technique, we can effectively increase the number of minority class samples, bringing the data to a balanced state where the quantities of both classes are equal. This approach makes the training data more comprehensive and diverse, potentially enhancing the model’s ability to learn from the minority class while reducing its bias towards the majority class. Finally, after data preprocessing, we combine the dataset *D*′ with the generator Φ_*G*_ to create the balanced dataset *D*_balanced_. First, we use the generator Φ_*G*_ to generate synthetic data *G*(*H*, cond) based on the conditional vector cond and Perlin noise *H*. The balanced dataset *D*_balanced_ is defined as follows:

$$\begin{array}{c} D_{\text{balanced}}=D^{\prime }\cup G(H,\text{cond}) \end{array}$$ (29)

The second step is to retain the original training data without any processing. This is mainly to reduce the possibility of the model overfitting to the synthetic data. Therefore, by combining the preprocessed dataset *D*′ with the synthetic data generated by CTGAN, we obtain a balanced dataset *D*_balanced_.

Next, we input the original unbalanced data *D*′ and the synthesized balanced data *D*_balanced_ into the model for training. The CTGAN-DC model uses the same base classifiers and meta-model as the DC model.

$$\begin{array}{c} {\hat{y}}_{CTGAN-DC}=\text{DC}\left(D_{balanced}\right) \end{array}$$ (30)

We input the training data into each base classifier for training, then integrate the results of each base classifier as the input for the meta-model. Based on the test set, we obtain the prediction result ${\hat{y}}_{CTGAN-DC}$, as shown in Algorithm 2.

**Algorithm 2** Pseudo-code of Disease Classifier with CTGAN (CTGAN-DC)

1: **Input:** KD dataset $D^{\prime }=D_{1}^{\prime \prime }\cup D_{2}^{\prime \prime }$, Generator model Φ_*G*_

2: **Initialization:** Oversampling using CTGAN, base classifiers (XGBoost, AdaBoost, CatBoost)

3: **Output:** Predicted labels for the test set

4: ***func*** TrainCTGAN_DCModel(Dataset *D*′):

5:  Use CTGAN model to generate synthetic data *G*(*H*, cond)

    5.1 Generate noise vectors *h*_*j*_ from Multivariate Normal Distribution (MVN) with mean 0 and identity covariance matrix *I*, for *j* = 1 to *m*

    5.2 Generate synthetic data samples ${\tilde{r}}^{j}=G\left(h_{j},{\text{cond}}_{j}\right)$, where *G* is the generator model

6:  Create balanced dataset *D*_balanced_ = *D*′∪*G*(*H*, cond)

7:  Split *D*_balanced_ into 5 folds: *D*_balanced,1_, *D*_balanced,2_, *D*_balanced,3_, *D*_balanced,4_, *D*_balanced,5_

8:  Initialize base classifiers: *B* = {XGBoost, AdaBoost, CatBoost}

9:  **for** *k* = 1 to 5 **do**

10:    Train set $D_{\text{train}}^{\left(k\right)}=D_{\text{balanced}}\backslash D_{\text{balanced},k}$

11:    Validation set $D_{\text{val}}^{\left(k\right)}=D_{\text{balanced},k}$

12:   **foreach** base classifier *b* in *B* **do**

13:    Train classifier *b* using $D_{\text{train}}^{\left(k\right)}$

14:    Predict on validation set $D_{\text{val}}^{\left(k\right)}$ and store predicted probabilities

15:    Store probabilities ${\hat{y}}_{i}^{\left(k,b\right)}$ for each sample in validation set

16:   **end for**

17:  **end for**

18:  Combine probabilities from each fold to form final layer output ${\hat{y}}_{\text{CTGAN}}$

19:  Train meta-model using Logistic Regression with ${\hat{y}}_{\text{CTGAN}}$ as features

20:  **return** Trained CTGAN-DC model

21: **end func**

22: ***func*** PredictWithCTGAN_DCModel(Trained CTGAN-DC model, Test dataset *D*_2_):

23:  Predict with base classifiers and pass the probabilities to meta-model

24:  Obtain final predicted labels

25:  **return** Predicted labels for *D*_2_

26: **end func**

Additionally, the DC and CTGAN-DC model architectures proposed in this paper can be integrated using a voting method. This involves combining the two sets of prediction results, ${\hat{y}}_{DC,i}$ and ${\hat{y}}_{CTGAN-DC}$. Here, ${\hat{y}}_{DC,i}$ is the predicted probability of the *i*-th sample generated by the DC model, and ${\hat{y}}_{CTGAN-DC}$ is the predicted probability of the *i*-th sample generated by the CTGAN-DC model. As shown in Algorithm 3, the voting method can use OR Gate, XOR Gate, or AND Gate to select the optimal sensitivity and specificity for predicting the final result ${\hat{y}}_{Ensemble,i}$, which can be expressed as:

$$\begin{array}{c} {\hat{y}}_{DC,i}=\text{DC}\left(D^{\prime }\right) \end{array}$$ (31)

$$\begin{array}{c} {\hat{y}}_{Ensemble,i}=\{\begin{array}{cc} {\hat{y}}_{DC,i}\vee {\hat{y}}_{CTGAN-DC} & \text{if}\;\text{OR}\;\text{Gate}\\ {\hat{y}}_{DC,i}⊕{\hat{y}}_{CTGAN-DC} & \text{if}\;\text{XOR}\;\text{Gate}\\ {\hat{y}}_{DC,i}\wedge {\hat{y}}_{CTGAN-DC} & \text{if}\;\text{AND}\;\text{Gate} \end{array} \end{array}$$ (32)

**Algorithm 3 Pseudo-code of Ensemble Prediction (Voting Mechanism)**

1: **Input:** Predicted labels from DC or CTGAN-DC models

2: **Output:** Final predicted labels

3: ***func*** EnsemblePrediction(Predicted labels from DC or Predicted labels from CTGAN-DC):

4:  **for** each sample *i* in test set **do**

5:    Use Voting mechanism (e.g., OR Gate, AND Gate, XOR Gate) to combine ${\hat{y}}_{\text{DC},i}$ or ${\hat{y}}_{\text{CTGAN-DC},i}$

6:    Obtain final predicted label ${\hat{y}}_{\text{Ensemble},i}$

7:  **end for**

8:  **return** Final predicted labels

9: **end func**

## Descriptive statistical analysis of Kawasaki Disease data

This section presents descriptive statistics for the KD datasets sourced from Kaohsiung Chang Gung Memorial Hospital and Kaohsiung Medical University Chung-Ho Memorial Hospital. This study utilizes data from Kaohsiung Chang Gung Memorial Hospital as the test set and Kaohsiung Medical University Chung-Ho Memorial Hospital as the validation set for KD prediction analysis. The test set from Kaohsiung Chang Gung Memorial Hospital consists of 79,400 patient records, with 1,230 positive KD cases and 78,170 negative febrile cases, resulting in a significantly imbalanced sample ratio of 64.60%. In contrast, the validation set from Kaohsiung Medical University Chung-Ho Memorial Hospital comprises 1,582 patient records, including 62 positive KD cases and 1,520 negative febrile cases, with an observed sample ratio of 24.50%.

For gender distribution, data from both hospitals show a higher incidence of KD in males than in females. Kaohsiung Chang Gung Memorial Hospital reports 687 male and 455 female-positive KD cases, whereas Kaohsiung Medical University Chung-Ho Memorial Hospital reports 37 male and 25 female-positive KD cases. In terms of age distribution, KD cases from both hospitals are predominantly in the infant stage, especially among children in the 0-1 year age group. In the analysis, we used an independent samples t-test to calculate the confidence intervals (CIs) and p-values, as detailed in Table 2.

**Table 2:** Kawasaki disease dataset descriptive statistics.

Characteristics	Kaohsiung Chang Gung Memorial Hospital (N = 79,400)				Kaohsiung Medical University Chung-Ho Memorial Hospital (N = 1,582)			
	Children with KD (N = 1,230)	Febrile controls (N = 1,230)	Odds ratio (95% CI)	p-value	Children with KD (N = 62)	Febrile controls (N = 1,520)	Odds ratio (95% CI)	p-value
**Birth, mean(SD)**								
Age, y	1.2 (1.2)	1.8 (1.6)	0.603 (0.563-0.646)	<0.001	2.4 (2.5)	3.1 (1.9)	0.832 (0.696-0.995)	0.002
**Gender, No. (%)**								
Male	746 (60.7%)	44037 (56.3%)	0.555 (0.482-0.639)	0.002	40 (64.5%)	1020 (67.1%)	0.951 (0.504-1.797)	0.431
**Blood, mean(SD)**								
RBC, 10^6^/*μ*	4.3 (0.4)	4.5 (0.6)	0.197 (0.091-0.423)	<0.001	4.4 (0.5)	4.6 (0.5)	69.5 (1.66-2909.689)	0.042
WBC, 10^3^/*μ*	14.1 (5.2)	10.8 (5.9)	1.001 (0.988-1.014)	<0.001	13.6 (5.6)	10.1 (5.3)	0.947 (0.888-1.01)	<0.001
Hemoglobin, g/dL	11.1 (1.1)	12 (1.8)	1.44 (0.863-2.403)	<0.001	11.3 (1.2)	12.0 (1.1)	1.55 (0.898-2.805)	<0.001
Hematocrit, %	33.4 (3.1)	35.6 (4.9)	1.069 (0.883-1.295)	<0.001	34.2 (2.9)	35.8 (2.9)	0.057 (0.005-0.648)	<0.001
MCH, pg/cell	25.7 (2.3)	27.1 (3.5)	0.623 (0.541-0.719)	<0.001	25.7 (3.2)	26.5 (2.5)	2.258 (1.082-4.711)	0.007
MCHC, g/dL	33.3 (1.1)	33.6 (1.2)	0.954 (0.776-1.173)	<0.001	33.1 (1.3)	33.5 (1.1)	0.044 (0.003-0.58)	<0.001
RDW	13.4 (1.4)	14.1 (2.1)	0.665 (0.633-0.7)	<0.001	13.6 (1.4)	13.3 (1.7)	0.843 (0.655-1.084)	0.072
Platelets, 10^3^/*μ*L	350.1 (125.8)	294.2 (125)	1.002 (1.001-1.003)	<0.001	324.6 (119.1)	263.7 (90.7)	1.004 (1.001-1.008)	<0.001
Neutrophils-segments, %	56.3 (15.4)	49.3 (19.9)	1.186 (1.147-1.227)	<0.001	58.2 (18.0)	57.9 (19.3)	0.981 (0.924-1.04)	0.9
Neutrophils-bands, %	1.6 (3.2)	0.7 (2.1)	1.232 (1.182-1.283)	<0.001	4.8 (7.2)	2.8 (6.2)	0.957 (0.887-1.034)	0.007
Lymphocyte, %	32.1 (13.9)	38.3 (18.3)	1.156 (1.116-1.196)	<0.001	26.3 (13.8)	29.5 (16.6)	0.972 (0.913-1.035)	0.114
Monocyte, %	6.8 (3.4)	8.4 (4.3)	1.039 (0.998-1.081)	<0.001	6.8 (3.3)	8.1 (3.6)	0.934 (0.844-1.034)	0.004
Eosinophils, %	2.5 (2.7)	1.8 (2.9)	1.325 (1.275-1.376)	<0.001	2.5 (2.7)	0.8 (1.6)	1.2 (1.071-1.345)	<0.001
Basophils, %	0.2 (0.4)	0.3 (0.4)	1.175 (1.026-1.347)	0.006	0.2 (0.4)	0.3 (0.4)	0.851 (0.394-1.836)	0.345
AST, U/L	77.9 (117.1)	51 (130.6)	0.997 (0.997-0.998)	<0.001	84.0 (102.0)	42.5 (31.9)	0.991 (0.986-0.997)	<0.001
ALT, U/L	76.0 (108.3)	34.6 (78.9)	1.005 (1.004-1.006)	<0.001	91.0 (127.8)	21.0 (24.6)	1.021 (1.013-1.029)	<0.001
CRP, mg/dL	74.9 (61.5)	20.1 (32.8)	1.015 (1.014-1.017)	<0.001	79.9 (49.5)	23.4 (34.8)	1.022 (1.016-1.028)	<0.001
**Urine, mean(SD)**								
Urine WBC count in urine, count/hpf	54.3 (101.6)	49.9 (66.8)	0.992 (0.991-0.993)	0.023	11.3 (21.9)	4.5 (13.9)	0.986 (0.968-1.004)	<0.001
**Urine, No. (%)**								
Pyuria	526 (42.8%)	5416 (6.9%)	0.093 (0.079-0.108)	<0.001	22 (31.9%)	195 (10.2%)	2.604 (1.09-6.219)	<0.001

Descriptive Statistics of the Kaohsiung Chang Gung Memorial Hospital Test Dataset and Kaohsiung Medical University Chung-Ho Memorial Hospital Validation Dataset.

**Fig 2:**
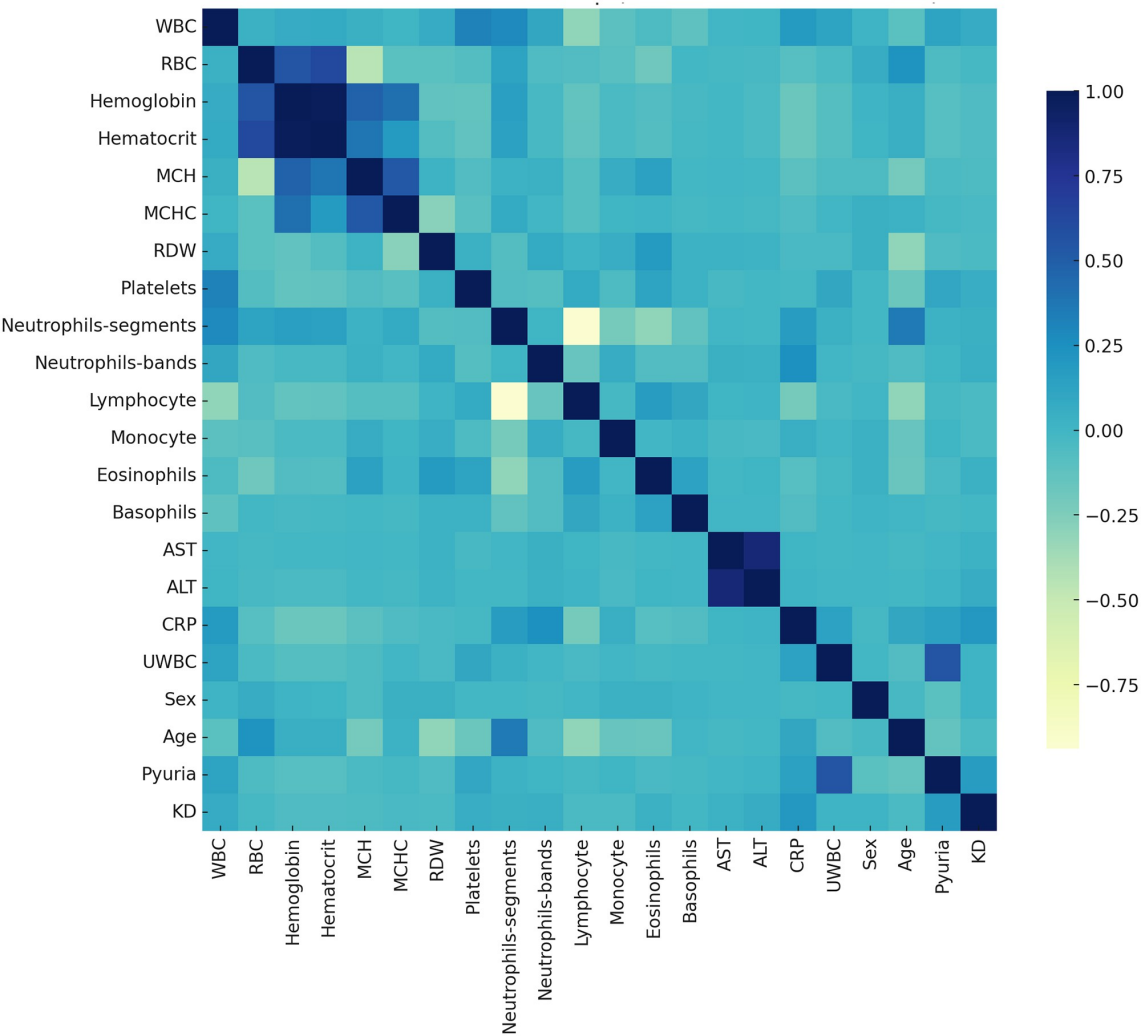
The Pearson correlation heatmap for variables in the Kaohsiung Chang Gung Memorial Hospital KD test set.

**Fig 3:**
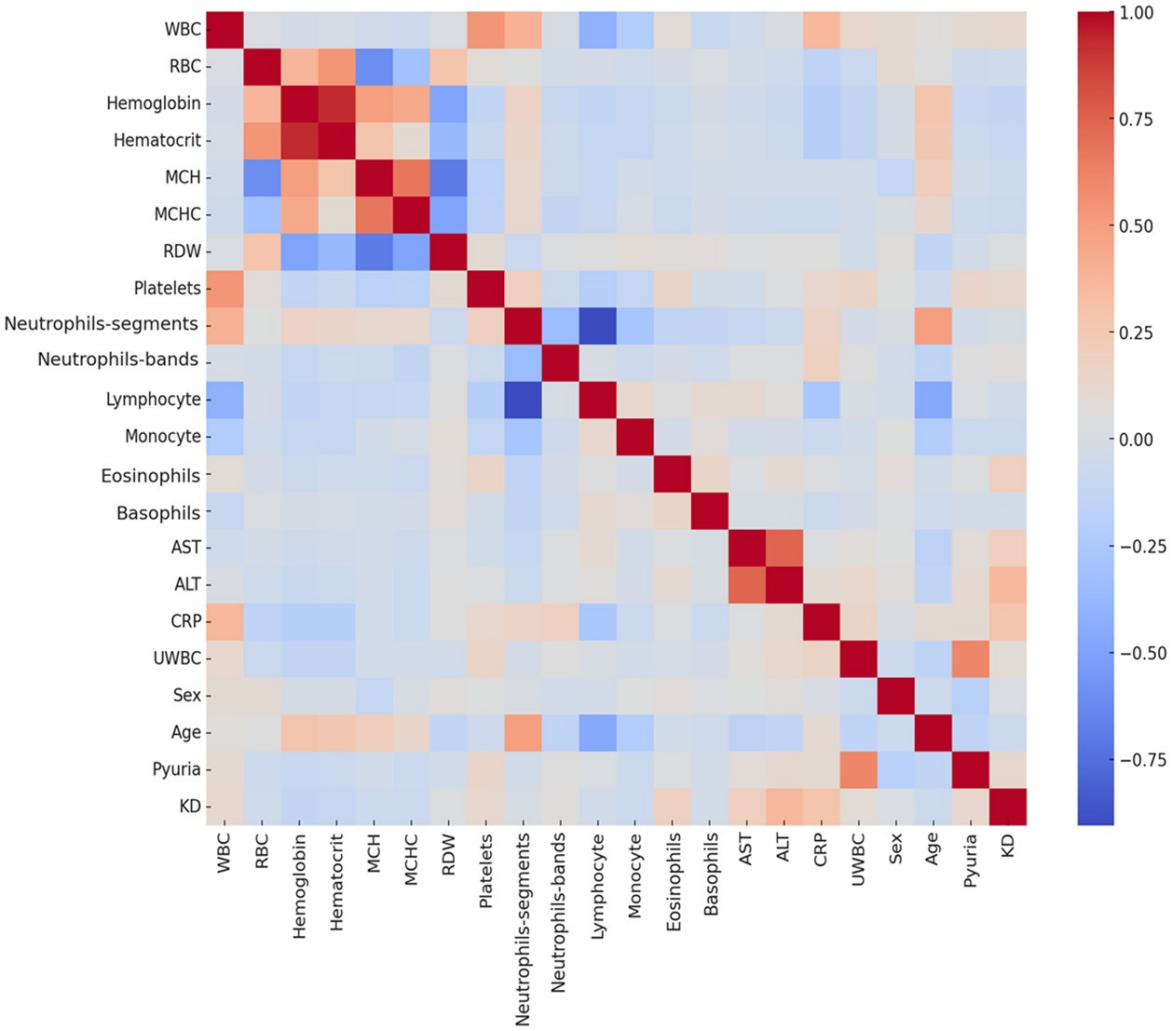
The Pearson correlation heatmap for variables in the Kaohsiung Medical University Chung-Ho Memorial Hospital KD validation set.

The Pearson correlation heatmap in Figs 2 and 3 illustrates the strength and direction of correlations among various clinical indicators. This visualization effectively reveals underlying correlation patterns within the data, enabling the identification of significant relationships between specific indicators. For example, a strong negative correlation is observed between neutrophils and lymphocytes, while C-reactive protein and erythrocyte sedimentation rate show a significant positive correlation. Such correlation analysis is essential for understanding interactions between variables and is critical for selecting or adjusting specific feature variables in model development.

## Results

The experiments were conducted on an Ubuntu 20.04 operating system, utilizing an AMD Ryzen 7 2700X CPU, an NVIDIA GeForce RTX 2080 GPU, and 32GB of memory, which offered substantial computational resources for the DC and CTGAN-DC models. This section provides a comprehensive account of the experimental results and analyses.

### Experimental methods

The Kaohsiung Chang Gung Memorial Hospital dataset consists of 79,400 records, divided into training and testing sets with an 80:20 split, maintaining proportional distributions of negative and positive cases. The training set includes 63,520 records, with 62,606 negative cases (febrile patients) and 914 positive cases (KD patients). The test set comprises 16,794 records, containing 16,566 negative cases and 228 positive cases.

A validation set from Kaohsiung Medical University Chung-Ho Memorial Hospital, consisting of 1,582 records (1,520 negative and 62 positive cases), was used to assess each model’s generalization capability. The models were evaluated using XGBoost in combination with CTGAN and TVAE for synthetic data generation, alongside the proposed DC and CTGAN-DC stacking approaches, to measure predictive accuracy and generalizability.

Training time for the CTGAN model is notably extended due to its complex data generation process, especially when addressing data imbalance. CTGAN requires additional time to generate a diverse range of synthetic data, prolonging the training phase. Similarly, the DC model’s stacked base classifiers increase training duration through cross-validation. In this study, training comprised 30,000 iterations, with the DC model requiring 4.2 hours and CTGAN-DC taking 6 hours to complete. Prediction time varies by model complexity and data batch size, averaging 10 milliseconds per instance for both CTGAN-DC and DC models.

### Experimental analysis

#### Evaluating data similarity in CTGAN-augmented datasets using Jensen–Shannon Divergence

To evaluate the fidelity of CTGAN-generated data, we used Jensen-Shannon Divergence (JSD), a metric that ranges from 0 to 1, with values closer to 0 indicating a higher similarity between the generated data distribution and the real data. This method enabled a comprehensive assessment of CTGAN’s effectiveness in replicating key features within Kawasaki Disease (KD) data. In our initial findings, critical features such as hemoglobin, hematocrit, and platelets had JSD values of 0.195, 0.204, and 0.195, respectively, demonstrating a high degree of similarity between the distributions of generated and real KD data. Additionally, JSD values for clinical indicators such as C-reactive protein (CRP) and urinary white blood cells (UWBC) were 0.162 and 0.142, supporting the generated data’s reliability in accurately representing clinical measurements.

When examining demographic features, gender (0.086) and age (0.187) also displayed low JSD values, underscoring CTGAN’s accuracy in replicating these demographic characteristics. However, the JSD values for red blood cells (RBC) and mean corpuscular hemoglobin (MCH) were somewhat higher at 0.404 and 0.433. While these values are elevated, they remain within an acceptable range, which may reflect the challenges CTGAN encounters in precisely modeling these specific features. An average mean JSD of 0.256 suggests that CTGAN-generated data effectively mirrors the distributional characteristics of real KD data, providing adequate reliability for further analysis and application.

#### Prediction performance on the Kawasaki Disease test set from Kaohsiung Chang Gung Memorial Hospital

**Fig 4:**
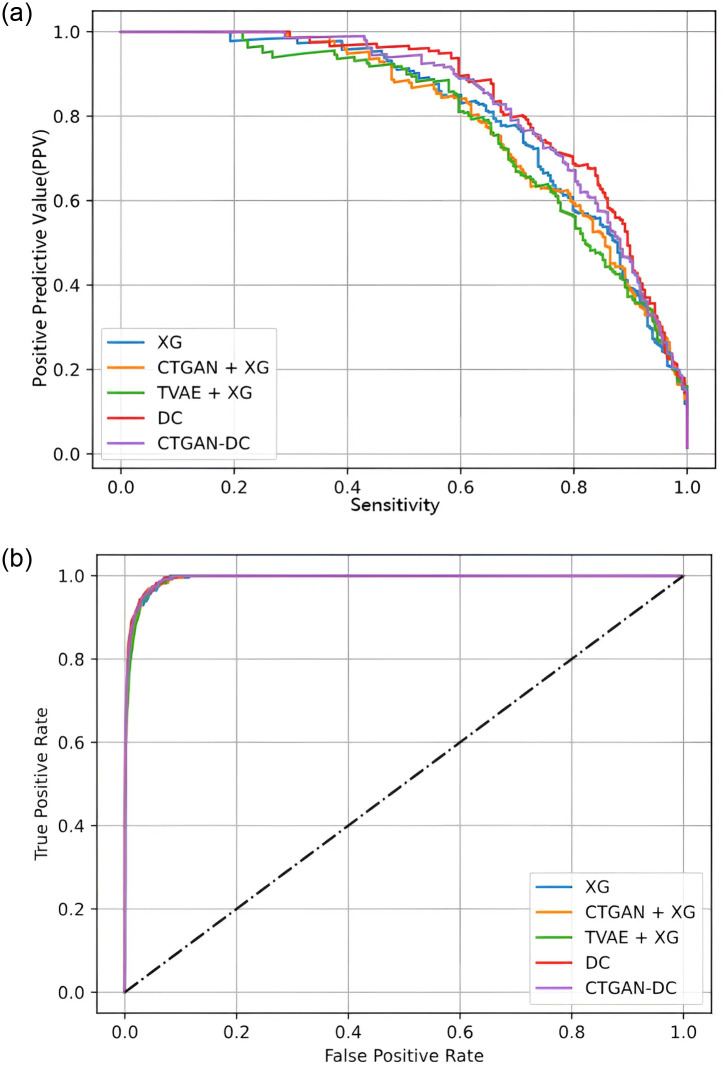
Prediction results of Kaohsiung Chang Gung Memorial Hospital KD test set. (a) PPV-Sensitivity curve. (b) ROC curve.

The experimental data in Table 3 show that the DC model consistently outperforms other models across various evaluation metrics under different Sensitivity conditions. When Sensitivity is at 90.4%, the DC method maintains a PPV of 47.8%, which is at least 4.7% higher than other models at the same Sensitivity level. However, under a Sensitivity of 95.2%, CTGAN-DC achieves the best performance in every model. This demonstrates that the DC and CTGAN-DC methods proposed in this study effectively reduce the number of false positives and exhibit better stability at higher Sensitivity levels. In Fig 4, (a) presents the PPV-Sensitivity curve, depicting the relationship between PPV and Sensitivity for each model and the method proposed in this study. The graph shows that as Sensitivity increases, the PPV of DC and CTGAN-DC at each stage consistently outperforms other models. The (b) displays the ROC curve.

**Table 3:** A comparative analysis of DC, CTGAN-DC, XGBoost, CTGAN-XG, and TVAE-XG models in Kawasaki Disease experiments.

Models/ Sensitivity	Kaohsiung Chang Gung Memorial Hospital KD Test Set				Kaohsiung Medical University Chung-Ho Memorial Hospital KD Validation Set			
	> 80%	> 85%	> 90%	> 95%	> 80%	> 85%	> 90%	> 95%
**XGBoost**								
Sensitivity	84.7	86	92.5	100	82.3	100	100	100
Specificity	99	98.8	97.3	0.03	86.2	0	0	0
Positive Predictive Value	55.6	53.3	34.5	0.02	19.5	3.92	3.92	3.92
Negative Predictive Value	99.8	99.8	99.9	100	99.2	-	-	-
**CTGAN-XG**								
Sensitivity	81.1	85.1	90.8	96.1	80.6	85.5	91.9	100
Specificity	99.1	98.8	97.6	96.1	91	86.7	78.1	0
Positive Predictive Value	58.9	51.7	37.1	27.7	26.7	20.8	14.6	3.92
Negative Predictive Value	99.7	99.8	99.9	99.9	99.1	99.3	99.6	-
**TVAE-XG**								
Sensitivity	80.3	85.5	90.8	96.1	80.6	100	100	100
Specificity	99	98.4	97.5	95.5	86.8	0	0	0
Positive Predictive Value	56.3	45.5	36.4	25	19.9	3.92	3.92	3.92
Negative Predictive Value	99.7	99.8	99.9	99.9	99.1	-	-	-
**DC**								
Sensitivity	84.2	85.5	90.4	95.2	80.6	85.5	90.3	96.8
Specificity	99.3	99.2	98.5	95.1	91.5	89	81.1	45.3
Positive Predictive Value	66.4	61.7	47.8	22.5	27.9	24.1	16.3	6.72
Negative Predictive Value	99.8	99.8	99.8	99.9	99.1	99.3	99.5	99.7
**CTGAN-DC**								
Sensitivity	83.3	85.5	90.4	**95.2**	80.6	85.6	90.3	**95.2**
Specificity	99.2	99	98.6	**97.0**	93.7	98.3	82.4	**95.0**
Positive Predictive Value	60.7	56.4	49.3	**32.3**	34.2	45.3	17.3	**24.1**
Negative Predictive Value	99.7	99.8	99.8	**99.9**	99.2	99.8	99.5	**99.9**

Using the Kaohsiung Chang Gung Memorial Hospital test dataset and the Kaohsiung Medical University Chung-Ho Memorial Hospital validation dataset, the study compared the predictive performance and generalization capabilities of the DC, CTGAN-DC, XGBoost, CTGAN-XG, and TVAE-XG models for KD.

#### Prediction performance on the Kawasaki Disease Validation Set from Kaohsiung Medical University Chung-Ho Memorial Hospital

**Fig 5:**
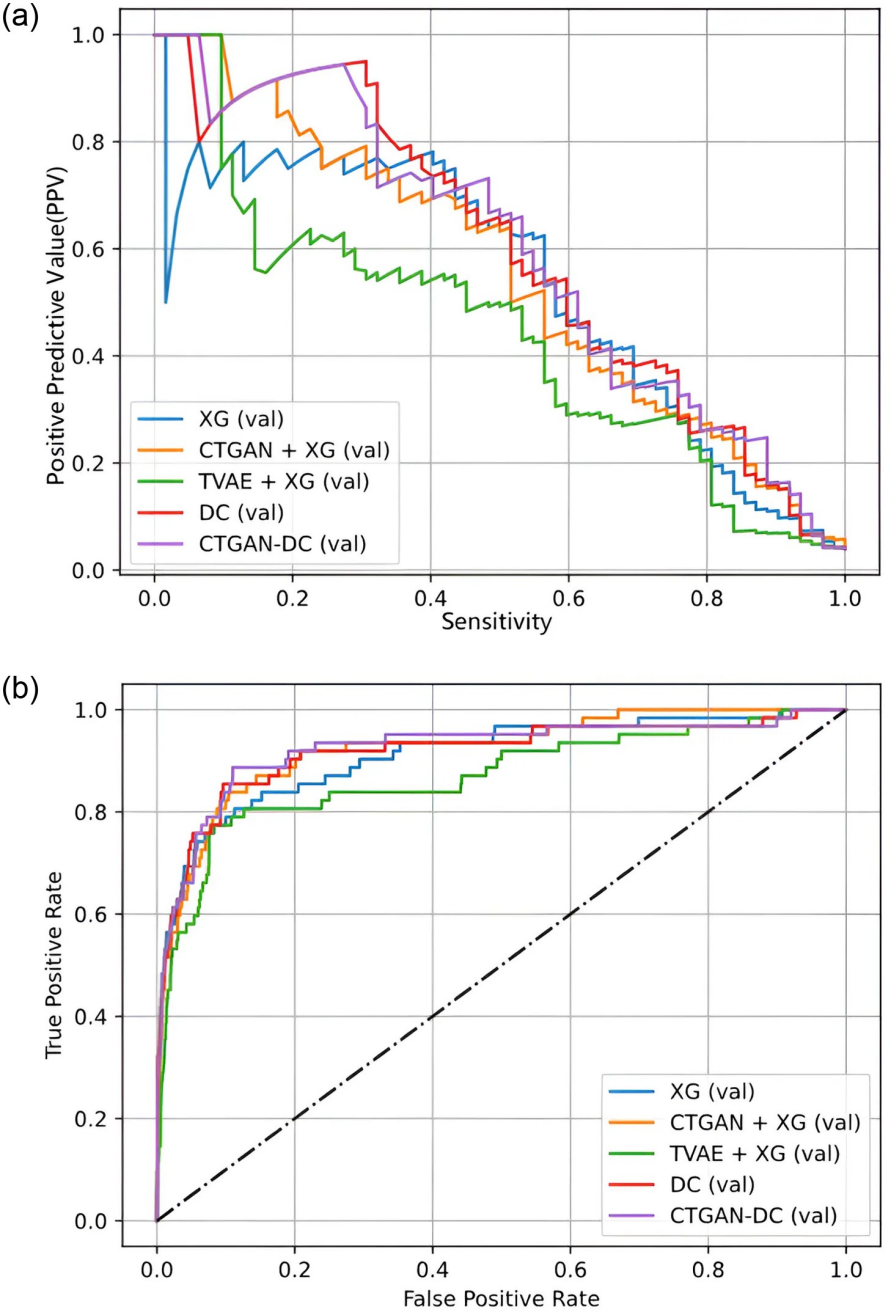
Prediction results of Kaohsiung Medical University Chung-Ho Memorial Hospital KD validation set. (a) PPV-Sensitivity curve. (b) ROC curve.

This study divided the validation data into two parts to demonstrate the generalization of the DC and CTGAN-DC methods. First, Table 3 used validation data from Kaohsiung Medical University Chung-Ho Memorial Hospital to test the models. The aim was to determine if the training data from Kaohsiung Chang Gung Memorial Hospital could equally distinguish between children with Kawasaki disease and febrile children. The experimental results show that the models perform less effectively on Kaohsiung Medical University Chung-Ho Memorial Hospital data than Kaohsiung Chang Gung Memorial Hospital—some models, including XGBoost, CTGAN-XG, and TVAE-XG, even demonstrating Model Collapse. However, the DC and CTGAN-DC methods still achieved Specificity levels of 81.1% and 95%, respectively, and Sensitivity levels of 90% and 95.2%, respectively, demonstrating outstanding overall performance, as shown in Fig 5.

#### Developing innovative DC and CTGAN-DC architectures to enhance accuracy and generalization in KD predictions

Table 4 shows that the proposed DC and CTGAN-DC architectures significantly improve the accuracy and generalization of KD model predictions through unique ensemble learning, oversampling, and stacking methods. Table 4 highlights three significant contributions and innovations. Firstly, the sensitivity and specificity of both DC and CTGAN-DC exceed those of methods in previous literature, with DC achieving 95% sensitivity and specificity and CTGAN-DC achieving 95% sensitivity and 97% specificity. Secondly, the proposed DC and CTGAN-DC models exhibit superior generalization capabilities compared to previous methods. In generalization tests, DC achieved 90% sensitivity and 81% specificity, while CTGAN-DC achieved 95% sensitivity and 95% specificity. The best previous KD prediction model [4] experienced model collapse during generalization testing, indicating a lack of consideration for generalization capabilities, which poses challenges in clinical use. Finally, the CTGAN-DC model demonstrates better generalization ability than the DC model, with a 14% improvement in specificity. This shows that CTGAN-DC effectively enhances the generalization capability of KD models through oversampling.

**Table 4:** Comparing the predictive performance and generalization capabilities of the DC and CTGAN-DC models with literature for KD Using sensitivity and specificity.

Models	Study Population	KD Datasets	Predictive Effectiveness in the Literature		KD Datasets	Validation Effectiveness in Kaohsiung Medical University Chung-Ho Memorial Hospital KD Dataset	
		*N* (Positive, Negative)	Sensitivity	Specificity	*N* (Positive, Negative)	Sensitivity	Specificity
Maki et al., 2018 [42]	Japan	129 (37, 92)	86%	86%	-	-	-
Lam et al., 2022 [30]	USA	1,517 (775, 742)	92%	95%	-	-	-
Wang et al., 2022 [43]	-	2,035 (1023, 1012)	80%	85%	-	-	-
Li et al., 2023 [31]	China	608 (299, 309)	86%	84%	-	-	-
Portman et al., 2023 [32]	USA	150 (50, 100)	92%	86%	-	-	-
Fabi et al., 2023 [44]	Italy	90 (34, 56)	98%	83%	-	-	-
Tasi et al., 2023 [4]	Taiwan	74,641 (1142, 73,499)	92%	97%	1,582 (62, 1,520)	100%	0%
DC	Taiwan	79,400 (1230, 78,170)	95%	95%	1,582 (62, 1,520)	90%	81%
CTGAN-DC	Taiwan	158,800 (79,400, 79,400)	**95%**	**97%**	3,040 (1520, 1,520)	**95%**	**95%**

In the experimental [30–32, 42–44], no models or scoring systems were provided, preventing an evaluation of their generalization using the Kaohsiung Medical University Chung-Ho Memorial Hospital KD Dataset. However, the study [4] employed the well-known XGBoost algorithm for the KD model, achieving the best sensitivity and specificity in predicting KD among previous studies. Therefore, comparing [4] with DC and CTGAN-DC adequately demonstrates the generalization performance of DC and CTGAN-DC, particularly CTGAN-DC.

## Discussion

This study introduces a Disease Classifier (DC) and the CTGAN-DC model, which achieve notable improvements in Kawasaki Disease prediction. This model significantly enhances prediction accuracy and generalizability by employing ensemble learning, oversampling, and stacking techniques. Additionally, the CTGAN-DC model’s data oversampling technique effectively balances the representation of minority classes, improving the model’s ability to learn from underrepresented data and reducing majority class bias. This approach enables the model to achieve high sensitivity and specificity across diverse clinical environments, underscoring its potential in real-world applications. Notably, testing datasets from two hospitals demonstrated the CTGAN-DC model’s robust generalizability, achieving high accuracy even under varied generalization conditions, confirming its strong clinical applicability.

Furthermore, the proposed ensemble learning framework offers a novel solution to data imbalance by combining the strengths of multiple algorithms. This approach enhances both stability and generalizability, proving especially effective in managing rare conditions like Kawasaki Disease.

To deepen understanding of the model’s decision-making process, this study applies explainable AI (XAI) techniques, specifically SHAP (SHapley Additive exPlanations), to comprehensively analyze prediction outcomes. Each key feature’s influence on predictions is examined in detail, with the Kawasaki Disease dataset serving as an illustrative example. SHAP values reveal that C-reactive protein (CRP) and white blood cell (WBC) counts are the most influential features, contributing SHAP values of 0.25 and 0.18, respectively. Moreover, platelet count and red blood cell distribution width (RDW) demonstrate significant impacts, with SHAP values of 0.12 and 0.10, respectively. These SHAP analysis results confirm the model’s capacity to accurately identify critical clinical features and assess each feature’s contribution to model decisions. This analysis enhances the model’s transparency, providing medical professionals with a more interpretable and supportive foundation for diagnosis.

**Fig 6:**
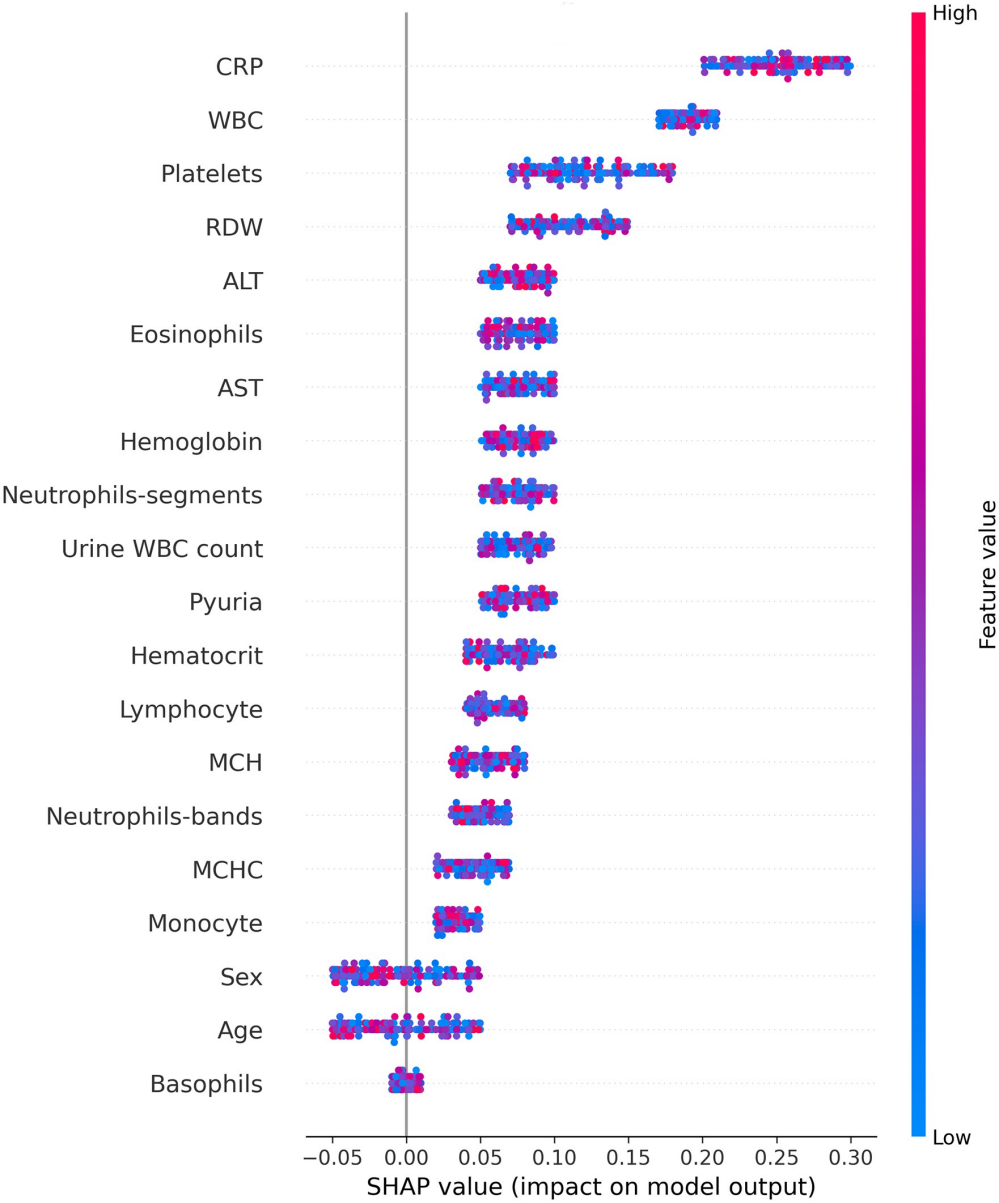
Explainable AI feature importance analysis of CTGAN-DC model for Kawasaki Disease prediction using SHapley Additive exPlanations (SHAP).

During testing with a Kaohsiung Chang Gung Memorial Hospital dataset, SHAP analysis showed that SHAP values for C-reactive protein (CRP) ranged from 0.2 to 0.3, indicating the feature’s significant influence on model predictions. SHAP values for both white blood cell (WBC) count and platelet count ranged from 0.1 to 0.2, underscoring the relevance of these features, as shown in Fig 6.

Finally, future research should explore refined feature selection strategies. Relying primarily on blood test data, due to challenges in collecting urine samples from children, may further enhance the model’s applicability across various clinical settings. Integrating additional potential features could further improve both predictive accuracy and generalizability.

## Limitation

This study introduces a disease classifier (DC) and a CTGAN-DC model for predicting Kawasaki Disease (KD), though certain limitations remain. Key challenges include the following:

Firstly, data imbalance poses a significant obstacle, even with Conditional Generative Adversarial Networks (CTGAN) used to address the skewed distribution of KD and febrile patient data. Synthetic data may still fail to capture subtle clinical variations, underscoring the need for more diverse data from a broader range of healthcare institutions to validate the model’s robustness across various populations and clinical settings.

Secondly, external validation has been limited to two major hospitals in Taiwan. Although the model performs well in these settings, its efficacy may vary internationally due to differences in clinical practices, patient demographics, and diagnostic standards. Expanding the validation through multi-center studies would be crucial to confirm the model’s generalizability.

Another limitation involves feature selection, which relies primarily on blood and urine test results. Given the challenge of collecting urine samples from young children, this dependency could restrict the model’s usability in clinical environments where urine samples are less accessible. Emphasizing features derived from blood tests could improve the model’s applicability across diverse healthcare settings.

Lastly, the CTGAN-DC model’s computational demands are substantial. While its accuracy and generalization capabilities are robust, the complexity of the model may hinder the training process in resource-limited environments. Future research should consider simplifying the model’s architecture to enhance training efficiency without compromising performance.

## Conclusion

This paper presents three key findings. First, the proposed DC and CTGAN-DC methods effectively predict Kawasaki Disease (KD), achieving the highest accuracy as validated by experimental results. Second, these methods significantly outperform existing models by attaining high sensitivity while maintaining superior specificity and positive predictive value (PPV) compared to previous studies. The DC model achieved 95% sensitivity and specificity, with generalization showing a 90% sensitivity improvement and an 81% specificity enhancement. In comparison, the CTGAN-DC model attained 95% sensitivity and 97% specificity, reflecting a 3% sensitivity increase. Additionally, the CTGAN-DC model’s generalization capabilities surpassed those in other studies by 95% for both sensitivity and specificity metrics. To further boost PPV, this study addresses critical challenges such as optimizing feature selection to prioritize blood test data over urine tests, balancing sensitivity and specificity to reduce false positives, and reinforcing validation through hybrid synthetic data generation. Future research will refine feature selection, focusing on blood tests alone to improve diagnostic efficiency, particularly for infants and young children. Incorporating additional potential features may also further enhance the model’s accuracy and generalizability.
